# Modalities of Exercise Training in Patients with Extracorporeal Membrane Oxygenation Support

**DOI:** 10.3390/jcdd9020034

**Published:** 2022-01-20

**Authors:** Christos Kourek, Serafim Nanas, Anastasia Kotanidou, Vasiliki Raidou, Maria Dimopoulou, Stamatis Adamopoulos, Andreas Karabinis, Stavros Dimopoulos

**Affiliations:** 1 Clinical Ergospirometry, Exercise & Rehabilitation Laboratory, 1st Critical Care Medicine Department, Evangelismos Hospital, National and Kapodistrian University of Athens, 106 76 Athens, Greece; chris.kourek.92@gmail.com (C.K.); sernanas@gmail.com (S.N.); a-icu@med.uoa.gr (A.K.); vraidou@gmail.com (V.R.); magiad@hotmail.com (M.D.); 2 Heart Failure and Transplant Unit, Onassis Cardiac Surgery Center, 176 74 Athens, Greece; stamatis.adamo@gmail.com; 3 Cardiac Surgery Intensive Care Unit, Onassis Cardiac Surgery Center, 176 74 Athens, Greece; karabinis291@hotmail.com

**Keywords:** extracorporeal membrane oxygenation (ECMO), intensive care unit-acquired weakness (ICU-AW), rehabilitation, exercise training, early mobilization, assessment

## Abstract

The aim of this qualitative systematic review is to summarize and analyze the different modalities of exercise training and its potential effects in patients on extracorporeal membrane oxygenation (ECMO) support. ECMO is an outbreaking, life-saving technology of the last decades which is being used as a gold standard treatment in patients with severe cardiac, respiratory or combined cardiorespiratory failure. Critically ill patients on ECMO very often present intensive care unit-acquired weakness (ICU-AW); thus, leading to decreased exercise capacity and increased mortality rates. Early mobilization and physical therapy have been proven to be safe and feasible in critically ill patients on ECMO, either as a bridge to lung/heart transplantation or as a bridge to recovery. Rehabilitation has beneficial effects from the early stages in the ICU, resulting in the prevention of ICU-AW, and a decrease in episodes of delirium, the duration of mechanical ventilation, ICU and hospital length of stay, and mortality rates. It also improves functional ability, exercise capacity, and quality of life. Rehabilitation requires a very careful, multi-disciplinary approach from a highly specialized team from different specialties. Initial risk assessment and screening, with appropriate physical therapy planning and exercise monitoring in patients receiving ECMO therapy are crucial factors for achieving treatment goals. However, more randomized controlled trials are required in order to establish more appropriate individualized exercise training protocols.

## 1. Introduction

Extracorporeal membrane oxygenation (ECMO) is an outbreaking, life-saving technology of the last decades which is being used as a gold standard treatment in patients with severe cardiac, respiratory or combined cardiorespiratory failure. ECMO support service is an advanced critical care procedure which should be implemented only by ECMO specialists in high-volume ECMO centers, providing maximum safety and efficacy to the very severe intensive care unit (ICU) critically ill patients. Despite optimal ECMO support management, patients suffer from several ICU-related complications, including ICU-acquired muscle weakness (ICU-AW). ECMO patients very often present ICU-AW as a complication mainly due to the high disease burden and prolonged ICU stay. ICU-AW is characterized by bilateral symmetrical limb weakness and/or neuromuscular weakness leading to persistent functional disability and decreased quality of life after hospital discharge [1].

Physical rehabilitation and early mobilization in critically ill patients have been shown to prevent and reduce ICU-AW burden, improve functional capacity, and at the same time decrease delirium, duration of mechanical ventilation, ICU and hospital length of stay and mortality rate, while improving post-ICU quality of life in critically ill survivors [2,3,4,5]. Despite the overall beneficial effects in critically ill patients, the role of physical rehabilitation in ECMO patients requires further analytic approach in order to be applied broadly in this very severe critically ill ECMO population. 

The aim of this qualitative systematic review is to summarize and analyze the different modalities of exercise training and its potential effects in patients on ECMO support, as well as to propose exercise protocols for different subgroups. 

## 2. Extracorporeal Membrane Oxygenation (ECMO)

ECMO is an Extracorporeal Life Support (ECLS) temporary therapy for patients with severe cardiopulmonary failure. The use of ECMO has increased rapidly during the last decade [6]. ECMO consists of a centrifugal pump, an oxygenator connected with a mixing gas (air–oxygen) blender, a heat exchanger providing blood from an inflow venous cannula to an outflow venous or arterial cannula for respiratory and circulatory support, respectively [7].

There are two basic types of ECMO: venoarterial (VA) and venovenous (VV). Both types of ECMO require two large cannulas. One cannula drains non-oxygenated blood from the venous system to the ECMO circuit through the centrifugal pump, and most specifically to the oxygenator, whereas the other cannula returns oxygenated blood back to either venous system (VV ECMO) or the arterial system (VA ECMO). Therefore, the main difference between VV and VA ECMO is that VA ECMO can be used mainly for heart support, whereas VV ECMO is used only for lung support [7,8]. Usually, the specialized ECMO team is responsible for ECMO configuration, the selection of VA or VV ECMO, and the type of cannulas needed [8]. A double-lumen cannula positioned through the jugular vein is an alternative ECMO configuration for potential candidates of lung transplantation allowing ambulation.

ECMO is considered in cases of refractory hypoxic and/or hypercapnia respiratory failure due to primary or secondary causes refractory to conventional mechanical ventilation, and refractory cardiogenic shock/cardiac arrest [9]. VV ECMO is used to support lung function, in cases of: (i) acute respiratory distress syndrome (ARDS) including severe bacterial or viral pneumonia, aspiration syndromes or alveolar proteinosis; (ii) extracorporeal assistance to provide lung rest in severe airway obstruction, pulmonary contusion or smoke inhalation; (iii) as a bridge to lung transplantation or perioperatively particularly for primary graft dysfunction support; (iv) status asthmaticus; (v) pulmonary haemorrhage or massive haemoptysis; and (vi) congenital diaphragmatic hernia, meconium aspiration [9]. On the other hand, VA ECMO is used in cases of refractory low cardiac output (cardiac index less 2 L/min/m^2^) and hypotension (systolic blood pressure < 90 mm·Hg) with signs of deteriorating tissue perfusion such as: (i) cardiogenic shock; (ii) severe cardiac failure due to acute coronary syndrome, cardiac arrhythmic storm, sepsis and drug overdose ⁄ toxicity with profound cardiac depression, myocarditis, pulmonary embolism, cardiac trauma or acute anaphylaxis; (iii) inability to wean from cardiopulmonary bypass after cardiac surgery; (iv) primary graft failure after heart or heart–lung transplantation; (v) chronic cardiomyopathy, either as a bridge to longer term VAD support or as a bridge to decision; (vi) periprocedural support for high-risk percutaneous cardiac interventions; and (vii), as a bridging method to transplant [9].

Absolute ECMO contraindications include: (i) unrecoverable heart and not a candidate for transplant or destination therapy of VAD support; (ii) disseminated malignancy; (iii) known severe brain injury; (iv) unwitnessed cardiac arrest; (v) prolonged cardiopulmonary resuscitation (>60 min) without adequate tissue perfusion; (vi) unrepaired aortic dissection; (vii) severe aortic regurgitation; (viii) severe chronic organ dysfunction (emphysema, cirrhosis, renal failure); (ix) compliance (financial, cognitive, psychiatric, or social limitations in patient without social support); (x) peripheral vascular disease for peripheral VA ECMO; and (xi) cardiogenic failure and severe chronic pulmonary hypertension (mean pulmonary artery pressure >50 mmHg) for VV ECMO [10]. Relative contraindications concerns factors such as advanced age (>75 years), obesity (>120 kg) or any contraindications for anticoagulation [9].

The ECMO beneficial effects in patients with severe acute respiratory failure have been shown in many studies during the last decade [11,12,13,14,15]. ECMO could be an effective cost-effective methodology of support in adults with severe ARDS [11,16]. A meta-analysis that combined data from two randomized controlled trials (CESAR and EOLIA trials), showed a reduction in mortality at 90 days [17]. Higher survival from early ECMO (two days after onset) compared with late ECMO (six days after onset) has also been shown [16]. ECMO has been used in the field of cardiac resuscitation [18,19], cardiogenic shock [20], pregnancy [10], as a bridge to heart transplantation, and even ARDS due to COVID-19 [21,22] showing a beneficial outcome.

ECMO is a complex methodology, considered a high-risk procedure with important complications such as bleeding events due to heparin [23,24], systemic thromboembolism [8] and severe heparin-induced thrombocytopenia (HIT) and haemolysis [8,16].

## 3. Intensive Care Unit-Acquired Weakness (ICU-AW) and ECMO Support

### 3.1. Prevalence and Risk Factors of ICU-AW

Intensive care unit-acquired weakness (ICU-AW), defined as bilateral symmetrical limb weakness and/or neuromuscular weakness, is the clinical manifestation of critical illness polyneuromyopathy. It is a common complication of critically ill patients in the ICU, leading to persistent functional disability and decreased quality of life after hospital discharge [1]. Electrophysiologic evidence of neuromuscular dysfunction has been observed in approximately 50% of ICU patients with sepsis, multiorgan failure, or prolonged mechanical ventilation [25]. However, the incidence increases up to 100% in patients with both systemic inflammatory response syndrome/sepsis and multiorgan failure [26].

ICU-AW has been shown to occur early and rapidly within the first 10 days of the ICU admission, and it is associated with increased hospital length of stay and increased mortality rates [27,28,29]. More than 25% of ICU patients undergoing mechanical ventilation for 7 or more days present clinical weakness on awakening, while diaphragmatic atrophy can be seen 18 h after the onset of mechanical ventilation [30,31]. The onset of weakness could present very early: from day 2 in the ICU [26].

Factors such as hyperglycemia, systemic inflammatory response syndrome (SIRS), sepsis, multiorgan dysfunction, renal replacement therapy and catecholamine administration increase the risk for the development of ICU-AW, as shown in a systematic review that included 1421 ICU patients with neuromuscular dysfunction [25]. There was no association between ICU-AW and age, gender, severity of illness, or exposure to corticosteroids or neuromuscular blocking agents [25]. In another French study, De Jonghe et al. found that the female gender, the number of days with dysfunction of two or more organs, the duration of mechanical ventilation, and the administration of corticosteroids were independent predictors of ICU-AW [30]. Whether the above predictors of ICU-AW are valid for ECMO patients remains to be answered from future studies.

Post-intensive care syndrome (PICS) is a term used to describe impairments in physical function, mental health, and cognition in patients after ICU discharge, and remains a major emerging problem of critically ill patients. Physical impairments of survivors of the ICU include neuromuscular weakness, ICU-acquired weakness, and musculoskeletal pain [32,33,34]. 

It has been suggested that patients requiring ECMO may present worse muscle wasting and weakness compared with ICU patients of other etiology, but there is no robust literature to support this hypothesis. In a prospective cohort study, which assessed the change in quadriceps size of 25 ICU patients on ECMO using ultrasound measurement from baseline to day 10 and day 20, it was found that there was a significant reduction of 19% in rectus femoris cross-sectional area by day 10, and a reduction of 30.5% by day 20 [35]. Moreover, at day 20 there was a moderate correlation between total muscle thickness and ICU mobility scale and Medical Research Council (MRC) sum-score of muscle strength [35]. In another study by Bear DE et al., the initial condition of the skeletal muscle index of patients with ARDS was related to the duration of VV ECMO, and most specifically, low skeletal muscle index resulted in longer VV ECMO duration, while preserved initial skeletal muscle index was associated with better survival [36]. Finally, fatty muscle fraction through CT imaging seems to be a predicting biomarker of 1-year mortality and a promising muscle quality imaging biomarker in ECMO patients [37].

Adrenaline and conditions including sepsis and multiple organ failure may contribute to the development of ICU-AW in critically ill patients receiving ECMO. Adrenaline produces the most significant skeletal muscle toxicity among all adrenergic agents [38]. Incidence of neuromuscular dysfunction increases up to 100% in ICU patients with both systemic inflammatory response syndrome/sepsis and multiorgan failure [26]. Nerve injury, skeletal muscle mass reduction and decreased muscle strength are important pathophysiologic characteristics of ICU-AW [39]. Decreased muscle strength and skeletal muscle mass reduction may be due to increased loss of protein and decreased muscle protein synthesis because of malnutrition and rhabdomyolysis [39,40]. Other possible potential mechanisms of protein reduction may be through the ubiquitin–proteasome system and autography [41]. Moreover, critically ill patients on ECMO usually present failure of muscle regeneration due to the muscle satellite cell injury [42,43].

### 3.2. Clinical Significance of ICU-AW

It has been shown that 5 years after ICU discharge, all patients report subjective weakness and decreased exercise capacity compared with their condition before ICU admission [1]. Patients who received ECMO treatment are more likely to experience worse chronic pain, depression, anxiety, and post-traumatic stress disorder and a much slower return to usual activity, up to 3 years after hospitalization [44,45]. However, in a single-center study from France including ICU patients, no differences in cognitive function, anxiety, depression and post-traumatic stress were demonstrated between those who were treated with ECMO compared with those who did not receive ECMO, after a 2-year follow-up [46].

Herridge et al. studied the long-term outcomes of 109 patients of ARDS in Canada and found that patients were mainly young (median age, 45 years) and critically ill (median APACHE II score, 23), with prolonged mechanical ventilation (median duration, 21 days), and an extended length of stay in the ICU (median duration, 25 days), as well as in hospital (median duration, 48 days) [47]. In these 109 ARDS survivors, the lung function improved significantly within the first year after ICU discharge, with normalization of lung volumes and spirometry by 6 months, and improvements in carbon monoxide diffusion capacity to 72% predicted at 12 months [47]. However, 1 year after ICU discharge, all patients reported poor function due to muscle bulk, proximal weakness, and fatigue, as observed in the six-minute walking test (6 MWT), where the median distance was 66% of the predicted [47].

The majority of critically ill patients usually return to work (77%) after ICU and/or ECMO discharge, but they often require a modified work schedule, gradual transition back to work, or job retraining [1]. Patients face psychologic ramifications of their severe illness, with more than 50% of survivors experiencing at least one episode of physician-confirmed depression or anxiety [1].

## 4. Rehabilitation in ECMO Patients—Safety, Feasibility and Efficacy

Physiotherapy and rehabilitation are essential needs for ECMO patients with severe cardiac and respiratory failure. ECMO patients are characterized by severe respiratory and/or cardiac failure which can severely affect and compromise peripheral tissues with significant functional impairment. Early mobilization and exercise in ECMO patients might represent a modality of training that could lead in the prevention of bed-rest-related problems, optimization of early recovery, and improvement in functional ability, mood and psychology [48].

Rehabilitation in critically ill ICU patients has been proven to be safe, and it has beneficial effects from the early stages in the ICU, resulting in the prevention of post-intensive care syndrome (PICS) and better outcomes regarding ICU-AW. Specifically, rehabilitation decreases episodes of delirium, durations of mechanical ventilation, ICU and hospital length of stay, and mortality rates. It also improves functional ability, exercise capacity, and quality of life [2,3,4,5]. A systematic review by Tipping C et al. [49] in 1753 patients from 14 studies demonstrated that although active mobilization and rehabilitation had no impact on short- or long-term mortality, it led to greater muscle strength at ICU discharge—as measured using the Medical Research Council (MRC) sum-score—greater probability of walking without assistance (activity limitation) at hospital discharge, and more days alive out of hospital at 6 months (participation restriction). Moreover, there were no consistent effects on function, quality of life, ICU or hospital length of stay, duration of mechanical ventilation or discharge destination [49].

Patients on ECMO are usually heavily sedated with prolonged bed rest and length of stay in the ICU, and are treated with muscle relaxants and corticosteroids which deteriorate muscle weakness and atrophy. Hayes K. et al. [50] investigated the early physical function and leg complications in patients who received ECMO before or after lung transplantation. They found out that strength and mobility levels were poor at ICU discharge (MRC score 44 ± 10, indicating ICU-AW), but both significantly improved by hospital discharge (*p* < 0.001) remaining, however, below normal (mean improvement MRC 11, 95% CI: 7–15, *p* < 0.001). Leg complications, including vascular and sensory neurological injuries, were reported in half of the survivors. In addition, ECMO survivors had a longer ICU (*p* < 0.001) and hospital stay (*p* = 0.002) and had worse physical function (6 MWT) than lung transplant recipients who did not require ECMO (*p* = 0.004) [50]. Moreover, ECMO patients may present complications because of the complexity of the system and the risk of cannula dislocation limiting exercise training. In order to minimize ECMO risks, rehabilitation in patients receiving ECMO therapy requires a very careful, multi-disciplinary approach, including a highly specialized team from different specialties, such as doctors, nurses, and physiotherapists [51,52]. Rehabilitation within the ICU should be applied to patients receiving ECMO after a thorough risk assessment of physical and nonphysical morbidity; the assessment of rehabilitation needs should take place within 24 h of ICU admission and active therapy should then be started in order to achieve better outcomes [52,53,54,55,56,57,58,59,60,61,62,63,64,65,66,67,68,69,70,71,72,73,74,75,76,77]. The characteristics, main results, and complications of the main studies that investigated rehabilitation in ECMO patients are presented in Table 1.

There is growing existing evidence for ECMO rehabilitation showing its safety for patients having either venous or arterial femoral cannulation. The safety of mobilizing these patients out of bed has been reported in a cohort study of ECLS-supported patients with respiratory failure, and in a larger single-center cohort of patients receiving ECLS as a bridge to recovery without any cannula-associated complications [57,72]. 

A systemic review and meta-analysis by Nydahl P et al. [78], which included 7546 critically ill patients and 22,351 mobilization/rehabilitation sessions showed that patient mobilization and physical rehabilitation in the ICU appears safe. Only 583 reported potential safety events, for a cumulative incidence of 2.6%, such as oxygen desaturation, hemodynamic changes, and removal or dysfunction of intravascular catheter [78]. Other safety events were falls and cardiac arrests in a quite small rate [74]. Feasibility and safety of ECMO rehabilitation in the ICU have been also shown during the last decade as shown in Table 1.

### 4.1. Screening and Assessment for Exercise

**Figure 1:**
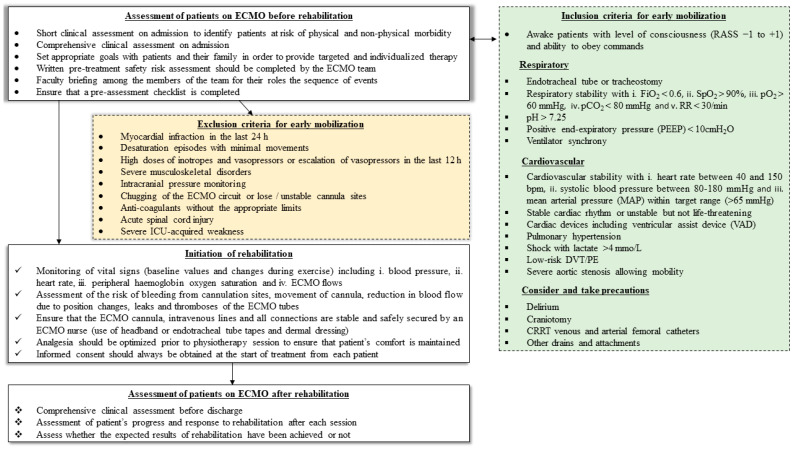
Screening and assessment of critically ill patients on ECMO support prior, during and after rehabilitation [48]. ECMO, extracorporeal membrane oxygenation; RASS, Richmond Agitation and Sedation Scale; RR, respiratory rate; DVT, deep vein thrombosis; PE, pulmonary embolism; ICU, intensive care unit.

Individualized rehabilitation has beneficial effects on every patient under ECMO support. Screening and assessment are very important factors in ECMO patients receiving rehabilitation (Figure 1). Almost all patients on ECMO are potential candidates for exercise and physiotherapy except for some special circumstances. Exceptions for receiving early rehabilitation are shown in Figure 1. VA ECMO patients require particularly thorough assessment prior to participation in rehabilitation sessions, due to the high risk of destabilization and the risk of peripheral arterial cannula dislocation. Pasrija C et al. [73] have shown that ambulating patients supported with VA ECMO, despite femoral arterial cannulation, is feasible and safe in carefully selected patients. There were no major bleeding events, vascular complications, or decannulation events associated with ambulation, and 1-year survival was 100% for ambulating patients. However, whether the application of mobilization has a positive impact on outcomes has yet to be proven. 

**Figure 2:**
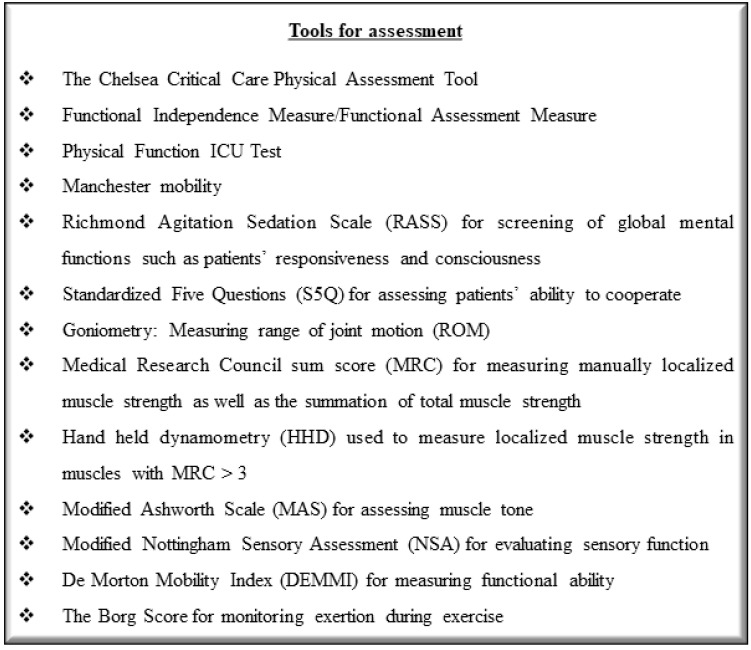
Tools for assessment of functional status in critically ill patients on ECMO support [79]. ECMO, extracorporeal membrane oxygenation; ICU, intensive care unit.

Exercise causes increased cardiac output, oxygen consumption and carbon dioxide production and, as a result, patients may require additional mechanical ventilation or ECMO support to perform a session of exercise [48]. Sometimes, the rehabilitation session should be terminated in case there are adverse changes in vital signs that do not respond to basic interventions [48]. It is essential for patients to be monitored even after the end of the session for delayed adverse responses to exercise [48]. Some proposed tools for the assessment of patients on ECMO performing exercise training are demonstrated in Figure 2.

### 4.2. Prehabilitation in ECMO Patients

“Prehabilitation” is a term used for rehabilitation in ECMO patients usually prior to heart or lung transplantation. Early mobilization has been shown to be beneficial in ECMO patients as a successful bridge to lung transplantation [80,81,82,83,84,85,86]. Although patients with acute cardiopulmonary failure managed without ECMO support have been more traditional candidates for mobilization [83], ECMO patients bridged to transplantation are candidates for rehabilitation as well. Patients receiving VV ECMO as a bridge to lung transplantation have been shown to be at greater risk for developing ICU-AW and being deactivated from the transplant list [50]. However, prehabilitation and increased physical activity while awaiting transplantation may prevent patients from deconditioning and help maintain transplant candidacy [87,88]. 

In a case report by Lowman et al. [54], a 16-year-old girl on ECMO with severe acute respiratory failure due to a cystic fibrosis exacerbation, received physical therapy interventions including therapeutic exercise, manual therapy, and integumentary protection techniques in addition to airway clearance techniques. Prior to her transplant, she was standing many times per day with moderate assistance, she was sitting on the edge of the bed, and she was taking steps to transfer from the bed to a chair, and vice versa. She successfully received a bilateral lung transplant after 8 days on ECMO. Turner DA et al. [53] presented two more cases, a 20-year old girl and a 24-year old girl with end-stage respiratory failure on ECMO awaiting lung transplantation. These patients performed active rehabilitation and physical therapy while they were still on ECMO. After their successful transplantation, the patients were liberated from mechanical ventilation, weaned to room air, transitioned out of the ICU, and became ambulatory in less than 7 days [53].

In an institution in New York, Biscotti et al. [84] investigated the effects of mobilization in 72 patients receiving ECMO as a bridge to lung transplantation within a 9-year period. Eventually, only 40 of them (55.6%) underwent the transplantation procedure. Within these 40 patients, 37 (92.5%) survived to discharge, and 21 (84.0%) survived for 2 years. The rate of inotropic or vasopressor support was significantly lower in those who underwent lung transplantation than those who did not (70% vs. 93.8%, respectively, *p* = 0.011), while the rate of ambulation was significantly higher (80% vs. 56.2%, respectively, *p* = 0.030). Finally, simplified Acute Physiology Score was significantly better in those who underwent lung transplantation (26.8 vs. 30.5, respectively, *p* = 0.048). Among the patients who were successfully transplanted but did not ambulate (*n* = 8), all patients were able to perform active physical therapy at the bedside and were on ECMO support for less than 7 days prior to transplantation. This study demonstrated favorable survival in patients receiving ECMO and early mobilization as a bridge to lung transplantation, and achieved high rates of physical therapy and avoidance of mechanical ventilation where ECMO was used in patients awaiting lung transplantation [84].

Although there is growing evidence of prehabilitation beneficial effects in ECMO patients awaiting heart or lung transplantation, there are still no randomized controlled trials comparing acute mobilization with usual care in these patients. Nevertheless, an awake and mobile ECLS strategy is safe and feasible in patients receiving ECMO support for advanced cardiac and respiratory failure [54,89]. Especially for patients awaiting transplantation, early mobilization should be a high priority [89].

### 4.3. Early Mobilization in the ICU

Early physical therapy in patients on ECMO in the ICU is strongly recommended because of its beneficial effects on muscle strength, physical function, health-related quality of life, ventilator-free days, and lengths of stay in both the hospital and the ICU (Table 1) [52,53,54,55,56,57,58,59,60,61,62,63,64,65,66,67,68,69,70,71,72,73,74,75,76,77]. An interesting systematic review by Ferreira DDC et al. [90] demonstrated that physical therapy, including early progressive mobilization, active-assisted exercises, sitting, standing, passive mobilization, resistance exercises, positioning in bed, stretching, and functional electrical stimulation of the lower limb muscles combined with cycling, together with ECMO, is feasible, relatively safe, and potentially beneficial for critically ill adult patients. Ko Y. et al. [63] studied the effects of early mobilization in eight ECMO patients in the ICU, seven with VV ECMO, and one with VA ECMO. A total of 62 sessions were performed, including 31 sessions (50%) of a passive range of motion and electrical muscle stimulation. Seventeen sessions (27.4%) were performed for patients who were sitting in bed or on the edge of bed, two sessions (3.2%) were for strengthening in sitting, eleven sessions (18%) were for standing or marching in place, while only one session (2%) was for walking. Eight sessions (13%) of sitting were supported with invasive mechanical ventilation. The blood flow rate of ECMO was higher during physical training than before (*p* = 0.013) but the sweep gas-flow rate of ECMO remained unchanged (*p* = 0.321). The main reasons for terminating a small rate (5%) of sessions were tachycardia and tachypnea.

The potential candidacy of early mobilization will be based on the underlying etiology and the intention of ECMO support. The use of an upper-body cannulation approach is usually preferred when mobility is of a sufficiently high priority [89]. Sommers J. et al. [79] edited a statement on rehabilitation recommendations in ECMO patients. In this statement, they suggest that rehabilitation management is determined primarily by the level of each patient’s consciousness. Thus, exercise interventions are divided into active interventions for patients who are able to follow instructions and make movements, and passive interventions for patients who are not able to follow instructions [79]. In patients who are conscious and able to follow instructions, a sequence of five active ranges of motion exercises is recommended daily for the prevention of joint contractures and muscle tone, while 1 to 3 sets of 8 to 10 repetitions of active exercises, as well as 20 min of active cycling, are recommended for the prevention of muscle atrophy and improvement of muscle strength [91,92,93,94,95]. In addition, mobilization in a standing position and walking, daily training and active cycling of 20 min per day are recommended for the improvement of their functional performance [91,92,93,95,96,97]. As far as the unconscious patients are concerned, passive mobilization should be performed on them. Stretching, splinting and passive movements using continuous passive motion should be applied for 20 min daily in order to avoid the risk of joint contractures [91,92,98]. Stimulation of muscle contractions is also very important and passive cycling for 20 min, continuous passive motion and electrical muscle stimulation should be applied daily [95,99,100,101,102]. Finally, respiratory rehabilitation is an important aspect of early mobilization in the ICU which helps patients to decrease ventilator-associated pneumonia (VAP), improves static lung compliance, enhances sputum clearance and reduces atelectasis [103].

It has been observed that muscle-building exercise programs with early mobilization help in rapid recovery by the prevention of critical illness polyneuropathy leading in the prevention and reverse of muscle wasting, which occurs early and rapidly during the first week of illness [29,103]. Regarding the potential impact of early mobilization on discharge rates of ECMO patients, two single-center studies of active physical therapy in patients with ECLS-supported cardiopulmonary failure, showed high rates of discharge to home or rehabilitation centers [57,72]. In the first study, Wells CL et al. [72] examined the feasibility and safety of mobilizing patients on ECMO support. The majority of patients (66.7%) received a total of 607 physical therapy sessions while still on ECMO support, and a percentage of 80.2% had at least one femoral cannula during physical therapy intervention. Among those who performed exercise, 68.8% were successfully weaned from ECMO and discharged to a rehabilitation facility with 23.8% returning home. The number of complications during exercise which interrupted the therapy sessions was low (< 0.5%), with mobility activities and exercises resuming normally the same day. Moreover, no major events were observed. Authors proved that it is feasible and safe to deliver early rehabilitation, including standing and ambulation, to patients on ECMO support, even in those with femoral cannulation sites with VA and VV ECMO [72]. In the other single center study, Abrams D et al. [57] performed a retrospective cohort study of 100 consecutive ICU patients receiving ECMO at a university hospital. Thirty-five percent of the 100 patients who were receiving ECMO performed active physical therapy consisting of 7.2 ± 6.5 sessions. More than half of them ambulated during physical therapy sessions, with a median distance of 175 feet. A large percentage of the bridge to recovery patients (88%) survived to discharge, with more than half of those discharged surviving to transplantation, and the great majority (90%) surviving to discharge after transplantation. Among the survivors, 57% went home, 35% went to acute rehabilitation, and 9% went to subacute rehabilitation. The important fact is that no complications related to physical therapy were reported [57].

Early mobility interventions have been proven to be beneficial in ECMO patients, even in the COVID-19 era. There is a characteristic case of a 27-year old pregnant woman who was infected with COVID-19 and admitted to the ICU due to the rapid development of acute respiratory failure [75]. Due to the severity of the respiratory failure, she was applied emergent ECMO cannulation, and placed in prone positioning. The patient was therefore referred for VV ECMO, where she remained for 9 days. Physical therapy interventions, such as in-bed active-assisted motion exercises, were held during days 2 to 4 because the patients could not perform exercise out of bed. Patients were eventually mobilized to sitting at the edge of the bed and standing during day 5, and out-of-bed activity was held on day 6. The young woman was able to achieve standing at the bedside with improved duration of stance, strength of standing force, and performance of pre-gait exercises on days 7 and 8 on VV ECMO. Supine exercises were also performed daily to ensure her level of consciousness and that there were appropriate responses in vital signs. On day 9, she was weaned out of the ECMO. She continued exercise interventions, including the progression of ambulation distance, the introduction of resistance band exercises, and the performance of single-stair repetitions; the frequency of her exercise sessions increased to twice per day. She was discharged on day 14 with outpatient physical therapy follow-up. The authors showed that early mobility while intubated on ECMO, and infected with COVID-19, is feasible while adhering to infectious disease precautions [75].

## 5. ECMO and Specific Exercise Interventions

### 5.1. Neuromuscular Electrical Stimulation (NMES)

Neuromuscular electrical stimulation (NMES) is an alternative type of mobilization in critically ill patients and patients on ECMO. It can be performed in both conscious patients and patients with a decreased level of consciousness. NMES has been shown to improve anatomical features and reverse pathophysiogical alterations, as it increases muscle thickness, improves microcirculation, increases oxygen consumption and reperfusion, and prevents patients from muscle atrophy [101,104,105,106]. Moreover, it has a beneficial effect on their functional status by increasing their muscle strength and preventing critical illness polyneuromyopathy [100,102,107]. Regarding other outcomes, NMES decreases the weaning time and the length of stay in the hospital and ICU [107,108].

There is only a single study regarding the application of NMES to the quadricep muscle in patients on VV ECMO [76]. In this study, four VV ECMO patients were applied a 30-min active NMES first, and a sham NMES session after, until a visible contraction of the quadriceps muscle and a palpable tensioning of the patellar ligament was experienced. NMES was well tolerated by patients and was shown to increase pedal blood flow; thus, providing a viable alternative to volitional exercise. Due to the limited data of NMES as an alternative way of exercise in ECMO patients, more randomized controlled trials are required.

### 5.2. Cycling

Cycling has been established as a supplementary type of early physical training. It can be applied either in conscious critically ill patients with better functional condition via a stationary bike out of bed, or in patients with deteriorated functional status through passive in-bed cycling. In a retrospective analysis by Hermens JA et al. [61], nine patients with end-stage lung disease on VV ECMO underwent immediate initiation of mobilization where a bed bike was included in the exercise program. Four were successfully bridged to lung transplantation, and five patients did not survive to transplant. In all surviving patients, mobilization improved lower body muscle strength. The mean MRC score before training was 3.75 (3–4), and the mean MRC score 1 day before the transplantation was 4.25 (4–5) [61].

In a case-series report, Rahimi RA et al. showed that active in-bed cycling is safe and feasible as a type of rehabilitation for patients on ECMO as a bridge to lung transplantation [55]. Most specifically, active in-bed cycling was beneficial for a critically ill 23-year-old patient with ARDS, a 37-year-old woman with pulmonary fibrosis and persistent right pneumothorax, and a 25-year-old woman with cystic fibrosis and severe obstructive pulmonary physiology. They all needed orthotopic lung transplantation; therefore, physical therapy and in-bed cycling helped them to obtain progressive, functional recovery preoperatively on ECMO support as a bridge to lung transplantation, and postoperatively [55].

In a case report from another institution, Pastva A et al. [65] performed FES cycling with a 30-year-old female patient on VV ECMO before and after lung transplantation. The patient performed two sessions before and five sessions after transplantation for over 18 days. Each session had a duration of 20–43 min, with a 3.62–6.89 miles distance and power of 1.59–8.96 watts. Muscle thickness of the rectus femoris and vastus intermedius was stable during the stay at the hospital, and increased to >2 cm from hospital discharge until outpatient rehabilitation completion. MRC sum-score at the ICU discharge was 58/60 and the handgrip strength was 60 pounds. Conclusively, muscle wasting was remarkably less compared with other instances referred to in the literature [65].

Finally, a worthwhile case report is the effective and feasible early rehabilitation in a 66-year-old woman with severe COVID-19 after bilateral lung transplantation [77]. The patient underwent a 30-min endurance training program on a bedside cycle ergometer with a moderate to slightly high intensity every day. She also performed breathing exercises, 6 to 10 repetition maximum weight training of the upper and lower limbs, airway clearance techniques, transfer training, daily therapeutic bronchoscopy and psychological support. The ECMO was successfully removed, and the individualized rehabilitation program improved her physical function, muscle strength, and quality of life, thus resulting in the patient’s good prognosis.

### 5.3. Strength Training

Strength training is an important rehabilitation tool of ICU-AW prevention and treatment in patients on ECMO support. Most exercise training protocols perform both aerobic exercise and strength endurance training [52,53,54,55,56,57,59,60,61,62,63,64,65,66,67,68,69,70,71,72,73,74,75,76,77]. Strength training exercises could be either passive in-bed for the early stages of prehabilitation and early mobilization, or active out-of-bed for conscious patients with better neuromuscular conditions and functional capacity. Passive or in-bed strength exercises include strengthening and reconditioning exercises in the supine position, such as ankle pumps, heel slides, upper extremity stretching, and a range of motion exercises [53], exercises in the sitting position on the edge of the bed to strengthen the torso, upper extremities, and lower extremities such as leg lifts, ankle rolls, and arm lifts [53], sitting in the bed or at the edge of bed, standing, functional electrical stimulation cycling in quadriceps, hamstrings, and buttocks bilaterally [65], and generally a various range of motion at the bedside [72,75,77]. On the other hand, active or out-of-bed strength exercises include muscle training of the lower extremities, such as dynamic quadriceps training by leg press, bed bike, or squats from sitting position and bed-to-chair mobilization [61], active exercises with elastic bands, mini-leg press [70], sit-to-stand transfer activities and functional strengthening using sit-to-stand from the bed or chair [72], stand–pivot transfers or taking small steps from the bed or chair with purpose to transfer to another surface [72], standing activities such as standing balance and tolerance, strengthening, weight shifting, marching, and stepping in place [72], active upper and lower limb and endurance exercises [74], or weight training of the upper limbs [77]. Intensity and the number of repetitions of strength exercises are usually individualized for each patient separately, according to his/her functional status and the stage of rehabilitation after evaluating maximum muscle strength of the muscle training group with one repetition maximum (1 RM) and/or handgrip and handheld dynamometers assessment.

### 5.4. Proposed Exercise Training Protocols per Subgroup of Patients

There are no established exercise training protocols for patients on ECMO in the literature. In our manuscript, we aim to propose modalities of exercise training for each subgroup of patients on ECMO support, suggesting exercise protocols for each modality and the potential candidates for each protocol (Table 2). Although it could be a guide for the future rehabilitation of critically ill patients on ECMO support, each protocol should be individualized according to each patient’s needs.

## 6. Clinical Implications and Contraindications for Rehabilitation in ECMO Patients

Although exercise seems to be safe and efficient in ECMO patients, there are some circumstances in which the risks of physical therapy likely outweigh the benefits. These include significant hemorrhage, severe arrhythmias, hemodynamic instability requiring moderate-to-high-dose vasopressors and severe hypoxemia or acidemia. Speculating from a recent meta-analysis by Nydahl P. et al. [78]—who has investigated 7546 critically ill patients and 22,351 rehabilitation sessions—583 safety events were reported with a cumulative incidence of 2.6%. The most frequently reported events were oxygen desaturation and hemodynamic changes, in 33 studies (69% of eligible studies), and removal or dysfunction of intravascular catheter in 31 studies (65% of eligible studies). Other potential safety incidents were 21 falls (in 43% of studies), 20 endotracheal tube removals (42%), 17 removals of intravascular catheters (35%), 23 other catheter or tube removals (48%) and 22 cardiac arrests (46%) [78].

Similar precautions should be taken into consideration during ECMO rehabilitation sessions. Moreover, exercise sessions should be discontinued if patients become unstable during activity. Clinically unstable patients may need to reconsider mobilization strategy or may be more appropriate for in-bed exercises or passive range of motion.

Another possible side effect of rehabilitation in patients on ECMO support could be dislocation of the ECMO cannulas, which may cause disturbance of the ECMO flow, decannulation, or severe bleeding. However, there are no studies in the literature to report such an event so far.

Absolute contraindications for out-of-bed rehabilitation are reported in Figure 1.

## 7. Future Perspectives

During the last decade, data regarding the early mobilization of critically ill patients on ECMO support have demonstrated its feasibility and safety with several beneficial effects in functional capacity, muscle strength and quality of life, leading to better prognosis and higher rates of hospital discharge. However, more observational and particularly randomized controlled studies are required in order to provide more robust evidence. Due to the ECMO methodology complexity and high-risk procedure, it is of paramount importance to form and validate individualized exercise training protocols for ECMO patients. Establishing individualized rehabilitation protocols could possibly lead to faster ECMO weaning, higher survival to hospital discharge rates, and a better functional status after heart or lung transplantation. An ECMO Physiotherapy Network registry at a national and international level can accumulate the current evidence interacting with the ECMO centers in order to disseminate updated ECMO rehabilitation knowledge.

Despite significant improvements in the ECMO devices, ECMO technology should focus creating more compact, portable, durable and patient friendly devices minimizing the associated risk during exercise and mobilization. Cannula dislocation during mobilization is a major concern for ECMO patients, significantly limiting exercise training in these patients. ECMO configurations with emphasis to secure cannula fixation should be developed; for example, the creation of a custom-made ECMO helmet that could achieve an optimal fixation while maintaining free and low-risk mobilization in patients with single- or double-lumen jugular cannulas [60].

## 8. Conclusions

Early mobilization and physical therapy have been shown to be safe, feasible and rather beneficial in patients with ECMO support. It is also beneficial and essential for patients receiving ECMO as a bridge to lung transplantation. Exercise training in ECMO patients should be conducted only at experienced ECMO centers under the direct supervision and collaboration of a highly specialized multidisciplinary team with well-defined roles and responsibilities. Initial risk assessment and screening with the appropriate physical therapy planning and exercise monitoring are crucial for achieving prevention and treatment goals in ECMO critically ill populations. More advanced exercise interventions such as NMES, cycling, or a combination, should be further investigated and tested in ECMO patients to ensure feasibility, safety, and efficacy.

## Figures and Tables

**Table 1:** Studies including mobilization in ECMO patients—sample size, intervention characteristics, outcomes, main results and complications.

Study	Sample	Intervention	Frequency and Duration	Outcomes	Main results	Complications
Turner DA et al., 2011, (Case series report)	Patient 1: with respiratory failure (cystic fibrosis) on VV-ECMO as a bridge to lung transplantation Patient 2: with end-stage cystic fibrosis and respiratory failure on VV-ECMO as a bridge to lung transplantation. Patient 3: with cystic fibrosis and respiratory failure due to influenza B infection on VV-ECMO as a bridge to lung transplantation	*In all patients*: – Strengthening and reconditioning exercises in the supine position (ankle pumps, heel slides, upper extremity stretching, and range of motion exercises) – Exercises in the sitting position – On the edge of the bed to strengthen the torso, upper extremities, and lower extremities (leg lifts, ankle rolls, and arm lifts) – Exercises in the standing position and ambulation	Patient 1 → 1 week Patient 2 → 1 week Patient 3 → 8 days	Lung transplantation	Successful bilateral orthotopic lung transplantation and weaning from ECMO	No rehabilitation-related complications
Lowman GD et al., 2012 (Case report)	1 patient with severe acute respiratory failure due to a cystic fibrosis exacerbation on VV-ECMO as a bridge to lung transplantation	– Therapeutic out-of-bed exercises (sitting on the edge-of-bed, steps to transfer to/from a chair) – Manual therapy – Integumentary protection techniques – Airway clearance techniques	9 days	Survival to lung transplantation	Successful bilateral orthotopic lung transplantation	No rehabilitation-related complications
Rahimi RA et al., 2013 (Case report study)	Patient 1: respiratory failure (worsening dyspnea and persistent right pneumothorax) on VV-ECMO as a bridge to lung transplantation Patient 2: respiratory failure (multidrug-resistant pneumonia) on VV-ECMO as a bridge to lung transplantation	– Supine therapeutic exercises – Active in-bed cycling – Sitting at the edge of the bed with assistance – 30 min of active, in-bed cycling	12 days 3 days	Lung transplantation Lung transplantation	Successful right orthotopic lung transplantation Successful bilateral orthotopic lung transplantation	No rehabilitation-related complications No rehabilitation-related complications
Rehder KJ et al., 2013 (Retrospective case series)	4 out of 9 patients with end-stage lung disease on VV-ECMO as a bridge to lung transplantation	Stretching and resisted exercises, sitting, standing, and ambulation (mean distance of 780 m)	5 days	Unknown	↓ in the mechanical ventilation time after transplantation ↓ in total length of hospital and ICU after transplantation 1-year survival 100%	No rehabilitation-related complications
Abrams D et al., 2014 (Retrospective cohort study)	35 out of 100 consecutive patients on ECMO underwent active physical therapy → - 19 of them as bridge to transplantation (16 on VV-ECMO and 3 on VA-ECMO) - 16 of them as bridge to recovery (15 on VV-ECMO and 1 on VA-ECMO)	– Bed-level active-assisted range of motion – Sitting in bed – Sitting at the edge of the bed – Standing – Ambulation	7.2 ± 6.5 sessions in total per patient 2.8 sessions per patient per week	Survival to transplantation or discharge Discharge disposition among survivors Critical safety events	Of the 16 bridge to recovery patients → 14 (88%) survived to discharge. 10 out of 19 bridge to transplantation patients (53%) survived to transplantation → 9 (90%) of these 10 patients survived to discharge Improvement in functional capacity Other: no overall difference in mean ECMO blood flow rates or sweep gas flow rates	No rehabilitation-related complications
Cork G et al., 2014 (Case report)	1 patient with severe respiratory failure due to Influenza A (H1N1) on VV-ECMO	Chest physiotherapy (positioning, ventilator hyperinflation, expiratory chest wall shaking and suctioning)	2 to 3 times daily for 13 days	Unknown	Successful weaning from ECMO support Improved secretion clearance and pulmonary recovery	Unknown
Morris K et al., 2014 (Case report)	A 46-year-old woman with acute viral interstitial pneumonia on VV-ECMO	- Passive mobilization - Bedside sitting - Active exercises	Unknown	Unknown	Desaturation during the intervention managed by increasing the blood flow in ECMO No other complications related to cannulation and normal cardiac response to exercise (increase in heart rate and systolic blood pressure)	
Pruijsten R et al., 2014 (Case report series study).	6 patients with respiratory failure on VV-ECMO	Exercises ranging room exercising in bed to walking outside the room	4–17 days	Unknown	No cannulas’ dislocation 4 patients successfully bridged to bilateral lung transplantation Other: 4 patients were able to stand and walk a short distance up to 100 m 2 patients could sit upright with both legs hanging outside of the bed	No rehabilitation-related complications
Hermens JAJM et al., 2015 (Retrospective analysis)	9 awake, non-intubated patients with end-stage lung disease on VV-ECMO as a bridge to lung transplantation	- Extensive sputum mobilization - Muscle training of the lower extremities (dynamic quadriceps training by leg press, bed bike or squats from sitting position) - Bed-to-chair mobilization	Unknown	Muscle strength (Medical Research Council -MRC)	↑ in lower body muscle strength [mean MRC from 3.75 (range 3–4) before training to 4.25 (range 4–5) 1 day before transplantation] Other: 4 patients successfully bridged to lung transplantation 5 patients did not survive to transplantation	- 1 patient with large rectus hematoma - 1 patient with obstructing thrombus in the return cannula
Kikukawa T et al., 2015 (Case report)	A 54-year-old man with H1N1 influenza-associated respiratory failure and severe obesity on VV-ECMO	Respiratory therapy and bedside sitting	3 days	Unknown	Improvement in respiratory function Successful decannulation on ICU 3 days after physical therapy Discharge from the ICU 5 days after physical therapy Discharged from the hospital with no severe disability	No rehabilitation-related complications
Ko Y et al., 2015 (Retrospective study)	8 patients on ECMO: - 7 patients on VV-ECMO → 2 of them as a bridge to recovery and 5 as a bridge to lung transplantation - 1 patient on VA-ECMO as a bridge to heart transplantation	- 31 sessions (50%) of passive range of motion and electrical muscle stimulation - 17 sessions (27.4%) sitting in bed or on the edge of bed - 2 sessions (3.2%) strengthening in sitting - 11 sessions (18%) standing or marching in place - 1 session (2%) walking	62 sessions	Safety events during physical therapy and interruptions due to unstable vital signs	Three sessions (5%) were stopped due to tachycardia (*n* = 1) and tachypnea (*n* = 2) during standing or marching in place Improvement in functionality and fitness	
Kulkarni T et al., 2015 (Case report)	A 36-year-old man with status asthmaticus on VV-ECMO	Active rehabilitation and ambulation (800 feet/day)	2 days	Unknown	Successful weaning from ECMO 2 days after rehabilitation Hospital discharge 5 days after rehabilitation	No rehabilitation-related complications
Pastva A et al., 2015 (Case report)	A 30-year-old woman with cystic fibrosis and respiratory failure due to severe pneumonia on VV-ECMO as a bridge to lung transplantation	- Functional electrical stimulation cycling in quadriceps, hamstrings, and buttocks bilaterally - Progressive mobilization	7 functional electrical stimulation sessions (2 pre and 5 post transplantation) for over 18 days	Efficacy of functional electrical stimulation before and after bilateral orthotopic lung transplantation	Maintenance of the muscle mass of the rectus femoris (1.5–1.6 cm) and vastus intermedius (0.95–1.15 cm) during hospitalization Increase in muscle mass after hospital discharge of more than 2 cm in both muscles Improvement in muscle strength at ICU discharge (MRC sum score of 58/60 and hand grip strength of 60 pounds)	No rehabilitation-related complications
**Bain JC et al., 2016** (Retrospective cohort analysis)	5 out of 9 patients with respiratory failure on VV-ECMO as a bridge to lung transplantation	Active physical rehabilitation and ambulation	Unknown	Economic impact of ambulatory versus non-ambulatory ECMO strategies	↓ by 22% in total hospital cost, by 73% in post-transplant ICU cost and by 11% in total cost in ambulatory ECMO patients compared with non-ambulatory ECMO subject Other: Lower length of mechanical ventilation before transplantation and higher ECMO support in ambulatory ECMO patients Shorter length of mechanical ventilation and ICU stay after transplantation in ambulatory ECMO patients	Unknown
Boling B et al., 2016 (Retrospective case series study)	18 patients with severe respiratory failure on VV-ECMO	- Physical therapy - Range of motion at the bedside - Ambulation in the hospital	Unknown	Unknown	8 patients (44%) received a transplant and survived to discharge Rest of the patients (10): 4 out o were successfully weaned from VV-ECMO 6 died	No rehabilitation-related complications
Keibun R. 2016 (Prospective observational study)	10 awake and 13 non-awake patients with refractory acute respiratory failure on VV-ECMO as a bridge to recovery (23 patients out of 31 who survived to ICU discharge)	Active rehabilitation	Unknown	Unknown	- Shorter length of stay in the ICU (13.6 vs. 21.7 days) in awake compared to non-awake ECMO patients - Shorter hospital stay (41.9 vs. 60.0 days) in awake compared to non-awake ECMO patients - Better improvement in the self-ambulation rate at discharge (70% vs. 38.5%) in awake compared to non-awake ECMO patients - Reduction in the total cost ($673,000 vs. $814,000) in awake compared to non-awake ECMO patients	Unknown
Norrenberg M et al., 2016 (Case series study)	10 patients with respiratory or cardiac failure on ECMO (5 on VV-ECMO and 5 on VA-ECMO)	Mobilization of all joints except for the limb used for ECMO cannulation	Unknown	Unknown	4 deaths (40%)	No rehabilitation-related complications
Munshi L et al., 2017 (Retrospective cohort study)	61 ARDS patients on ECMO out of 107 as a bridge to recovery (57 on VV-ECMO and 4 on VA-ECMO) → 50 patients of them underwent physiotherapy while 11 did not (47 on VV-ECMO and 3 on VA-ECMO)	- Passive ROM - Active ROM - Sitting - Standing - According to ICU Mobility Scale	Unknown	Association between ICU physiotherapy and ICU mortality Factors associated with a higher intensity activity score	Association with ICU mortality (*p* < 0.05): ICU physiotherapy (OR 0.19; 95% CI 0.04–0.98) APACHE II (OR 1.13; 95% CI 1.01–1.26) Sex (OR 8.4; 95% CI (1.71–41.7) No clinical characteristics were independently associated with the intensity of ICU physiotherapy except for SAS	No rehabilitation-related complications Only complications related to ECMO (such as barotrauma, limb ischemia, intracerebral hemorrhage, HIT, air embolism)
Salam S et al., 2017 (Case report)	A 50-year-old man with severe ARDS on VV-ECMO as a bridge to lung transplantation	- Active exercises with elastic bands - Mini-leg press - Bedside sitting - Ambulation	125 days	Lung transplantation	Improvement in fitness before lung transplantation Successful bilateral lung transplantation after 125 days on ECMO	Cannula fracture during ambulation
Shudo Y et al., 2018 (Case report)	1 patient on VA-ECMO while awaiting en-bloc heart-lung transplantation	- 45-degree tilt and 40% weight-bearing for 30 min in the first day - Full tilt and weight bearing for 30 min after 7 days - Step off and ambulate several feet at bedside with assistance after 10 days - Ambulation for 30 min with minimal assistance and strengthening exercises after 14 days	19 days	Unknown	Successful en-bloc heart-lung transplantation after 19 days Hospital discharge 12 days after transplantation	No rehabilitation-related complications
Wells CL et al., 2018 (Retrospective cohort study)	167 out of 254 patients on ECMO (98 on VV-ECMO and 69 on VA-ECMO)	- Therapeutic exercises (range of motion, stretching and strengthening exercises, muscle endurance, breathing exercises) - Bed mobility (rolling, supine to sit transfer training, bridging activities) - Edge of bed activities (sitting balance, posture, pre-standing activities, breathing and coughing) - Sit to stand transfer activities (sit to standing transfers and functional strengthening using sit to stand from the bed or chair) - Stand pivot transfers (pivot or taking small steps from the bed or chair with purpose to transfer to another surface) - Standing activities (standing balance and tolerance, strengthening, weight shifting, marching, and stepping in place) - Ambulation (gait training, gait speed, ambulation tolerance)	268 interventions 170 interventions 100 interventions 106 interventions 39 interventions 98 interventions 37 interventions	- Discharge disposition - Adverse events	109 survivors out of 167 patients (65%) VA ECMO: 41 out of 69 (59%) patients with hospital discharge VV ECMO: 68 out of 98 (69%) patients with hospital discharge	3 minor events (< 0.5%) → 2 episodes of arrhythmias (non-sustained ventricular tachycardia) and 1 hypotension event
Pasrija C et al., 2019 (Retrospective study)	15 out of 104 patients with decompensated heart failure and pulmonary embolism on VA-ECMO	- Motor strength exercises (moving to the chair or walking)	Unknown	Safety and feasibility of ambulation (absence of major bleeding, vascular, or decannulation events)	- 3 minor bleeding events (20%) - 100% in-hospital survival	
Braune S et al., 2020 (Prospective observational study)	43 out of 115 critically ill patients on ECLS with IMS ≥ 3 (12 on VV-ECMO, 17 on VA-ECMO, 7 on VV-ECCO_2_R, 3 on AV-ECCO_2_R and 4 on RVAD)	- Functional strengthening - Breathing exercises - Active upper and lower limb exercises - Endurance exercises - Progressing functional mobility	332 mobilizations (100 on VV-ECMO, 72 on VA-ECMO, 48 on VV-ECCO_2_R, 63 on AV-ECCO_2_R and 49 on RVAD) 130 min (IQR 44–215) median duration of all mobilization activities	Complications during mobilization	- 3 out of 43 (6.9%) patients with bleeding from cannulation site requiring transfusion and/or surgery - 1 episode (0.3%) of femoral cannula displacement during mobilization on VA-ECMO patient	
Mark A et al., 2020 (Case report)	1 pregnant woman with acute respiratory failure due to COVID-19 on VV-ECMO	- In-bed active-assisted range-of motion exercises such as sitting at the bedside and standing - Out-of-bed activities including standing at the bedside, strength of standing force, pregait exercises	6 days	Unknown	Successful hospital discharge	1 episode of hypotension (mild dyspnea with activity and lightheadedness)
McCormack PF et al., 2020 (Randomised crossover trial)	3 patients on VV-ECMO	30-min active NMES session delivered to the quadriceps (biphasic, symmetric impulses of 45 Hz, with 400 μs pulse duration, 12 s on and 6 s off) and 30-min sham session (intensity at the minimum value of 1–5 mA, without palpable contractions)	30 min/session	Pedal perfusion assessed via a combination of laser speckle contrast imaging (LSCI), non-imaging laser doppler (NILD) flowmetry, and transcutaneous oximetry (PtcO2)	Minimal change in pedal perfusion during NMES in 2 patients ↑ in pedal perfusion during NMES on the LSCI data in 1 patient	No rehabilitation-related complications
Mao L et al., 2021 (Case report)	1 patient with severe COVID-19 after bilateral lung transplantation on VV-ECMO	- Ventilation in the prone position → 12 h each day for 3 consecutive days - Ventilation in the supine position → 30 min each time, 4 times a day - Breathing training → 10 min each time, 2 times a day - Airway clearance technique → active breathing techniques once every hour - Exercise training → thoracic expansion exercises - Endurance training → bedside cycle ergometer for 20–30 min a day with moderate to slightly high intensity - Strength training → 6 to 10 RM weight training of the upper and lower limbs for 10–30 min, once or twice a day initially and 3 to 4 times a day later - Transfer training from the bed to the bedside, from the bedside to a chair, and from the chair to the bed repeatedly for 30 min each time, 2 times a day - Daily therapeutic bronchoscopy	2 days on ECMO (then ECMO was removed and the patient continued rehabilitation without ECMO)	Discharge from ECMO	Successfully removal of ECMO one day after rehabilitation	No rehabilitation-related complications

ICU, intensive care unit; IMS, intensive care unit mobility scale; ECMO, extracorporeal membrane oxygenation; VA, veno-arterial; VV, veno-venous; ECLS, extracorporeal life support; VV-ECCO_2_R, veno-venous extracorporeal carbon dioxide removal; AV-ECCO_2_R, arterio-venous extracorporeal carbon dioxide removal; RVAD, right ventricular assist device; NMES, neuromuscular electrical stimulation; HIT, heparin induced thrombocytopenia; SAS, sedation agitation scale; ROM, range of motion; RM, repetition maximum.

**Table 2:** Modalities of exercise training, proposed protocols, and suggested candidates.

Modality of Exercise	Proposed Exercise Protocol *	Suggested Candidates
**Prehabilitation**	- Airway clearance techniques - Ventilation in prone and supine position - In-bed active-assisted range-of motion exercises: Sitting at the bedside; Standing balance and tolerance; Rolling; Stretching; Positioning in bed; Strengthening and reconditioning exercises in the supine position including ankle pumps, heel slides and upper extremity stretching; Bedside cycling (passive/active) for 15 min (3 sets of 5–10 min with 1 min recovery); Inspiratory muscle training; Out-of-bed activities; Standing at the bedside; Strength of standing force; Sitting on the edge of the bed; Transfer from bed to chair; Exercises in the sitting position on the edge of the bed including leg lifts, ankle rolls, and arm lifts (strength training); Aerobic training (cycle ergometer/treadmill) for 10–20 min; Inspiratory muscle training. Proposed duration: 60 min per session, daily while on ECMO.	Patients with severe acute or end-stage respiratory failure on ECMO support as a bridge to lung transplantation. Patients with severe acute or end-stage heart failure on ECMO support as a bridge to heart transplantation.
**Early mobilization**	Awake patients with level of consciousness ➢ 1 to 3 sets of 8 to 10 repetitions of 5 active range of motion and resistance exercises such as leg press, squats from sitting position; ➢ 15–30 min of active cycling with moderate to slightly high intensity; ➢ Mobilization in a standing position and walking; ➢ Respiratory rehabilitation and breathing exercises for 2 sets of 10 min per set; ➢ Functional electrical stimulation of the lower limb muscles; Inspiratory muscle training. Unconscious patients ➢ Passive mobilization via stretching, splinting and passive movements; ➢ Continuous passive motion; ➢ Functional electrical stimulation of the lower limb muscles; ➢ Passive cycling for 20 min. Proposed duration: 60 min per session, 5 times per week while on ECMO.	Patients with severe acute or end-stage respiratory failure on ECMO support as a bridge to lung transplantation. Patients with severe acute or end-stage heart failure on ECMO support as a bridge to heart transplantation. Patients with respiratory or heart failure on ECMO support as a bridge to recovery.
**NMES**	- Functional electrical stimulation cycling in quadriceps, hamstrings, and buttocks bilaterally; - 30-min active NMES per session; - Biphasic, symmetric impulses of 45–75 Hz, with 300–400 μs pulse duration, 6 s on and 12 s off (intensity to a maximum of 140 mA). Proposed duration: 30 min per session, daily while on ECMO.	All patients on ECMO support, especially patients with lower level of consciousness.

* Selection of exercises (in-bed or out-of-bed, passive or active) should be individualized for each patient according to his/her consciousness level, functional status and disease severity. Workload, number of repetitions and number of sets in strength training depend on the physical status of each patient: 1 to 3 sets of 8 to 10 repetitions of 5 active range for patients with better physical status and 3 to 5 sets of 8 to 10 repetitions of 5 passive range for patients with worse physical status or disability for active rehabilitation is suggested. Workload should be based on the 1 RM test for active exercises (40–60% of the 1 RM test) if feasible. Moderate intensity of aerobic exercise training, either on a cycle ergometer or a treadmill, aimed for 12–13 on the Borg scale is suggested for prehabilitation or early mobilization in patients with higher severity and lower functional capacity while moderate to slightly high intensity is suggested for early mobilization in patients with better functional status. Inspiratory muscle training should be prescribed once or twice daily, with 3–5 sets of 6 inspiratory efforts at the intensity of 30–60% of maximum inspiratory pressure progressively increased. ECMO, extracorporeal membrane oxygenation; NMES, neuromuscular electrical stimulation; ICU-AW, intensive care unit acquired weakness; MAP, mean arterial pressure; SAP, systolic arterial pressure; HR, heart rate.

## Data Availability

Not applicable.
